# Prevalence of irregular menstruation according to socioeconomic status: A population-based nationwide cross-sectional study

**DOI:** 10.1371/journal.pone.0214071

**Published:** 2019-03-19

**Authors:** Yeunhee Kwak, Yoonjung Kim, Kyoung Ah Baek

**Affiliations:** 1 Red Cross College of Nursing, Chung-Ang University, Seoul, Korea; 2 Department of Nursing, Kookje College, Gyeonggi-do, Korea; Ordu University, TURKEY

## Abstract

Irregular menstruation is an important indicator of current and potential health problems. A woman’s health is greatly influenced by her socioeconomic status. The purpose of this study was to examine the prevalence of irregular menstruation by socioeconomic status among South Korean women. Secondary data analyses were conducted among 4,709 women, aged 19–54 years, using raw data from the Korea National Health and Nutrition Examination Survey V (2010–2012), a nationally representative survey. Compared to women who graduated from university, the adjusted odds ratios (95% confidence intervals) for those who graduated from elementary school or lower, middle school, and high school were 3.256 (1.969–5.385), 2.857 (1.866–4.376), and 1.607 (1.261–2.048), respectively. Compared to women with a medium-high income level, the adjusted odds ratio (95% confidence interval) for women with the highest household income level was 1.409 (1.091–1.819). Irregular menstruation was prevalent among adult women and appeared to be associated with socioeconomic status, especially in terms of education and household income. This study’s findings suggest that attention must be paid to women with low educational levels or high household incomes, to ensure early diagnosis and the provision of medical attention for irregular menstruation.

## Introduction

Irregular menstruation can have various health implications, and is an indicator of health in women [1]. The prevalence of irregular menstruation varies from 5% to 35.6% depending on age, occupation, and the country of residence [1,2,3,4]. In particular, the incidence of menstrual irregularity in adult Korean women is 14.3%; while this value is not high, it is increasing by 0.4% every year [5]. Irregular menstruation can result from hormone imbalances and stress; these factors act as both health indicators in women and as mediators of various health indicators. Irregular menstruation is related to mental health conditions, such as depression, in addition to physiological factors [6–8].

According to Statistics Korea 2015 [9], women have begun to enter the workforce at a later age owing to an increased educational level. Furthermore, they enter their first marriage and have their first child at average ages of 30 and 31.5 years, respectively. Various menstrual problems, such as amenorrhea, menstrual pain, and abnormal uterine bleeding occur especially frequently among women in their 20s–30s, and the incidence of these problems continues to increase after the age of 30 years [10]. Women with menstrual problems such as irregular menses, menorrhagia, amenorrhea, dysmenorrhea, and premenstrual symptoms report a significantly poorer health status [2,11]. Furthermore, the menstrual cycle is an indicator of general health in women [1,12]. A woman’s reproductive health status may affect her offspring’s health after birth [1,12]. Menstrual problems are considered important health indicators among working women, as an abnormal menstruation cycle is associated with health-related anxiety and dissatisfaction [13]. In addition, irregular menstruation has a negative effect on work productivity [2].

Menstrual cycle disturbances are a result of hormonal imbalances, which occur due to exposure to environmental stress, e.g., changes in energy balance (excessive physical activity, low energy intake), exposure to pollutants (present in polluted air and tobacco smoke), and psychosocial stress [8,12]. Menstrual irregularity, defined as an irregular menstrual cycle, is a form of abnormal menstruation that results from various causes, such as the presence of a disease (i.e., endometriosis, type 2 diabetes mellitus, etc.), medication use (i.e., drug-treated depression, antiandrogens, etc.), underweight or obesity, smoking habit, and reproductive factors (age at menarche, parity, etc.) [14–19]. Early diagnosis and treatment of menstrual irregularities can help reduce the occurrence rates of infertility and the sequelae of serious diseases such as congenital heart disease and osteoporosis [10,17–20]. However, South Korean women tend to have negative perceptions regarding visiting a gynecologist, and do not regard menstrual irregularity as an important health issue [20]. Furthermore, many women hope that their symptoms will subside over time and are often passive about receiving treatment [20]. Socioeconomic inequality is recognized as an important determinant of health and health behavior in adults [21,22]. Socioeconomic status can affect the development of chronic diseases such as diabetes, obesity and hypertension, and health behaviors such as smoking and eating habits, and participation in exercise [21,23–25]. Therefore, it is necessary to understand the relationship between socioeconomic status and irregular menstruation, as this can be an indicator of female health.

Researchers have observed differences in health status according to socioeconomic status [21,22] and associations between socioeconomic status, disease prevalence, and mortality [21,24,26]. Women with a low socioeconomic status have a poor nutritional status, indicating that socioeconomic status plays a crucial role in an individual’s health [27]. In a previous study, menstrual cycle length variations were more frequently observed with increasing age in women from lower social groups [28]. Manual workers and service/sales workers showed a higher frequency of irregular menstruation [21,29]. Lifestyle and psychological risk factors, such as smoking, obesity and stress were significantly associated with menstrual irregularity [30,31]. Stress levels and the mental health status of female college students were associated with irregular menstrual cycles and menstrual disorders [12] and menstrual irregularity was closely related to psychosocial health problems [32,33], depression [1] and short sleep duration [33]. There is a lack of research that directly analyzes the relationship between socioeconomic status and menstrual irregularity in women as a potential indicator of reproductive health. This study aimed to examine the differences in the prevalence of irregular menstruation according to demographic, socioeconomic, and health characteristics to clarify the relationship between irregular menstruation and socioeconomic status.

## Materials and methods

### Design and sample

The present investigation was a cross-sectional study based on secondary analyses of data from the weighted fifth Korea National Health and Nutrition Examination Survey (KNHANES V), which was conducted from 2010 to 2012.

The KNHANES has been performed since 1998 by the Korea Centers for Disease Control and Prevention (KCDC) for the evaluation of the health and nutritional statuses of Koreans. The KNHANES is a nationally representative, reliable, large-scale, cross-sectional survey targeting non-institutionalized Koreans. Sampling was conducted using a stratified, multi-staged, clustered probability design to ensure a nationally representative sample [34]. Furthermore, the KNHANES V adopted a survey sampling method that avoids duplication of the entire sample for large-scale sample surveys nationwide; therefore, the sample for every survey year was a probability sample representing the entire nation, and each sample has independent and homogenous characteristics. The KNHANES receives annual deliberation and approval from the Research Ethics Deliberation Committee of the KCDC, and all participants provide written informed consent. The health and nutrition surveys include one-on-one interviews and self-report questionnaires, while the clinical examination is performed by a specialized examination team at the KCDC, including nurses, physicians, nutritionists, and health science majors, whose investigative performance is verified via regular education and field quality control. The quality of the collected KNHANES data is overseen by the sub-department and mediation consult committee of the KCDC [34–36].

In the first year of the KNHANES V (2010), 8,958 of 10,938 people selected participated in the survey (survey participation rate, 81.9%) [34]. In the 2011 and 2012 KNHANES V, 8,518 of 10,589 people (survey participation rate, 80.4%) [35] and 8,057 of 10,589 people (survey participation rate, 80.0%), respectively, participated in the survey [36].

**Fig 1:**
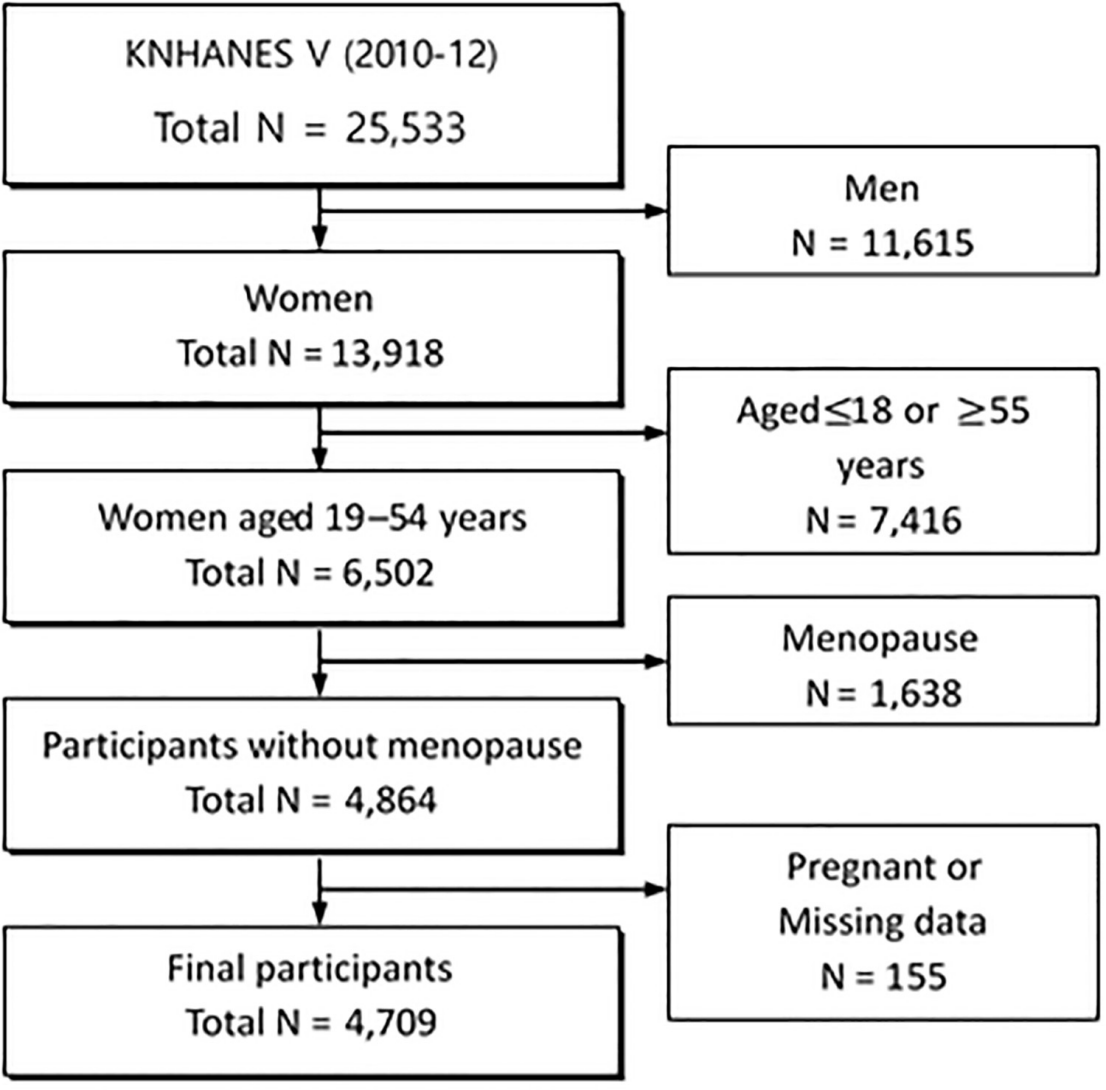
Flow chart of the study population. KNHANES, Korea National Health and Nutrition Examination Survey.

In this study, women aged 19–54 years who had not experienced menopause were selected from KNHANES V data (n = 25,533). We excluded data on 11,615 men and 7,416 women aged <19 years or >55 years. Of the remaining 6,502 women aged 19–54 years, 1,638 who had experienced menopause were excluded. Of 4,864 adult women, 155 were excluded because they were pregnant or had missing survey values; data on 4,709 women were used for the final analysis (Fig 1).

### Ethical approval

This study was approved by the Institutional Review Board of the KCDC (IRB No. 2010-02CON-21-C), and was performed in accordance with the ethical standards laid down in the 1964 Declaration of Helsinki and its later amendments.

### Measures

#### Irregular menstruation

Menstrual flow may occur in regular or irregular cycles [1]. In the health interview survey, participants were asked, “Is your menstruation regular?”; response choices were “yes” (regular menstrual cycle) or “no” (irregular). The responses were dichotomized for subsequent analyses.

#### Socioeconomic status

Information regarding participants’ socioeconomic status was obtained from questions on their educational level and monthly household income. Educational level was divided into elementary school graduate or lower, middle school graduate, high school graduate, and university graduate or higher. Household income was divided into quartiles based on the equivalent income (i.e., average monthly household income) and obtained by adjusting the household income for the number of household members; Q1, Q2, Q3, and Q4 represented the <25^th^, 25–50^th^, 50–75^th^, and >75^th^ percentiles of household incomes, respectively.

#### Covariates

We collected data on demographic and health-related variables including age, residence, spousal status, employment status, waist circumference (WC), body mass index (BMI), smoking status, drinking status, exercise level, stress status, age at menarche, and childbirth status. Residence area was divided into urban and rural. Employment status was categorized as currently employed *vs*. unemployed. Spousal status was defined as yes (i.e., currently living with a spouse or partner) or no (i.e., unmarried, divorced, not living with a spouse or partner, or widowed). WC was recorded to the nearest 0.1 cm at the midpoint between the lower part of the last palpable rib in the mid-axillary line and the upper part of the iliac crest at the end of expiration using a measuring tape (Seca 200; Seca). BMI was calculated by dividing the body weight (kg) by the square of the height (m^2^). Participants were categorized as current smokers *vs*. non-smokers, regardless of past smoking history. Alcohol consumption was calculated in grams per day based on the average number of alcoholic beverages consumed and the frequency of alcohol consumption. Participants who consumed alcohol at an average rate of 1–15 g/day, >15–30 g/day, and >30 g/day were considered mild, moderate, and heavy drinkers, respectively [37]. Regular exercise was defined as participation in ≥20 minutes/exercise session, ≥3 times/week [38]. Stress was based on the reported level of stress on an average day for each participant. Those reporting that they experienced “much” or “very much” stress were categorized as having high levels of stress (“yes”), while those who reported experiencing “little” stress were categorized as having low levels of stress (“no”). Age at menarche was defined as the age at first menstruation. Participants were also asked if they had ever experienced childbirth.

### Statistical analysis

All data are presented as means ± standard errors (SEs) for continuous variables or proportions ± SEs for categorical variables. The SAS survey procedure (ver. 9.3; SAS Institute Inc., Cary NC, USA) was used to run a complex sample design based on the design and nature of the survey data to obtain sampling weights for the KNHANES and nationally representative estimates. Differences in the prevalence of irregular menstruation according to the participants’ demographics, health-related characteristics, and socioeconomic status are presented as means or percentages ± SEs, and were verified via *t*-tests and χ^2^ tests. Educational level and household income were then combined into four groups for the assessment of group-specific differences in the prevalence of irregular menstruation via χ^2^ tests. Covariates (age, BMI, spousal status, WC, smoking status, drinking status, regular exercise, stress status, age at menarche, and childbirth status) were adjusted for in the multiple logistic regression analyses to evaluate the association between the prevalence of irregular menstruation and socioeconomic status by generating adjusted odds ratios (aORs) and 95% confidence intervals (CIs). In the multiple logistic regression analyses, clinically and statistically meaningful confounding variables were adjusted for, and a model was constructed. Model fit was assessed by the addition or removal of one variable at a time and comparison of the Akaike and Bayesian information criterion values. We evaluated the interaction between socioeconomic status and other variables in the analysis; however, no interaction terms were included in the final (current) model because they were not significant, based on a *p*-value <0.2. Statistical significance was set at a *p*-value <0.05.

## Results

### Irregular menstruation according to demographic and health-related characteristics

Of the women included in this study, 669 (prevalence, 14.2%) experienced irregular menstruation (Table 1). The average age of the participants was 35.4 years and 85.5% of them had an urban area of residence. A total of 79.1% of participants had husbands and 57.1% had an occupation. The mean WC was 74.9 cm and mean BMI 22.6. Most of the women did not smoke (7.4%), consume alcohol (3.8%) or participate in regular exercise (17.4%), while 36.1% of them said they were stressed. The average age at menarche was 13.8 years, and 65.6% of the participants had experienced childbirth. Most of the participants had a high school education or higher, and the household income was above medium-high in most cases.

**Table 1:** Prevalence of irregular menstruation according to demographic and health-related characteristics, and socioeconomic status (N = 4,709).

Characteristic	Total (n = 4,709) Mean ± SE or n (%)	Irregular menstruation		p-value
		No (n = 4,040) Mean ± SE or % (SE)	Yes (n = 669) Mean ± SE or % (SE)	
Age (years)	35.4(0.2)	35.5 ± 0.2	35.03 ± 0.5	0.366
Residence (urban)	4026(85.5)	86.5 (1.4)	84.6 (2.3)	0.276
Spousal status (yes)	3725(79.1)	82.3 (1.2)	75.9 (2.5)	0.004
Occupational status (yes)	2688(57.1)	58.3 (1.0)	55.9 (2.3)	0.330
Waist circumference (cm)	74.9(0.2)	74.6 ± 0.2	76.3 ± 0.5	0.001
Body mass index (kg/m^2^)	22.6(0.1)	22.5 ± 0.1	23.1 ± 0.2	0.001
Smoking status (current)	348(7.4)	6.0 (0.5)	8.7 (1.4)	0.028
Drinking status (heavy)	179(3.8)	3.4 (0.4)	4.2 (1.1)	0.392
Regular exercise (yes)	819(17.4)	17.1 (0.7)	17.7 (1.8)	0.738
Stress status (yes)	1699(36.1)	32.5 (0.9)	39.6 (2.2)	0.002
Age at menarche (years)	13.8(0.1)	13.8 ± 0.1	14.0 ± 0.1	0.030
Childbirth experience (yes)	3090(65.6)	66.8 (1.1)	58.7 (2.4)	0.001
Educational level				
≤Primary school graduate	136(2.9)	71.6 (4.3)	28.4 (4.3)	<0.001
Middle school graduate	246(5.2)	74.8 (3.2)	25.2 (3.2)	
High school graduate	2154(45.7)	83.2 (1.0)	16.8 (1.0)	
≥University graduate	2173(46.2)	88.8 (0.8)	11.2 (0.8)	
Household income				
Q1: lowest income	318(6.8)	84.6 (2.3)	15.4 (2.3)	0.170
Q2: medium-low income	1196(25.4)	85.7 (1.4)	14.3 (1.4)	
Q3: medium-high income	1564(33.2)	86.1 (1.1)	13.9 (1.1)	
Q4: highest income	1631(34.6)	82.5 (1.2)	17.5 (1.2)	

*Q*: quartile; *SE*: standard error

Women with irregular menstruation had higher WC values (p = 0.001), were likelier to have no children (p = 0.001), and had higher BMI values (p = 0.001) than those with regular menstruation. Furthermore, these women were likelier to be unmarried, divorced, not living with a spouse, or widowed (p = 0.004). Finally, women with irregular menstruation were likelier to be current smokers (p = 0.028), have higher stress levels (p = 0.002), and have an older age at menarche (p = 0.030) than those with regular menstruation.

### Irregular menstruation according to socioeconomic status

Of the socioeconomic variables, only the educational level was significantly different between women with and without regular menstruation (*p <*0.001; Table 1). Women with irregular menstruation had a lower educational level than those with regular menstruation.

Model 1 was adjusted for age and BMI; model 2 was additionally adjusted for smoking status, drinking status, regular exercise, and stress status; and model 3 included all the covariates in model 2 in addition to age at menarche and prior childbirth (Table 2). The reference groups for educational level and household income comprised those with a university education or higher and Q3 (medium-high income), respectively.

**Table 2:** Association between socioeconomic status and irregular menstruation (N = 4,709).

	Model 1^a^	Model 2^b^	Model 3^c^
**Educational level**			
≤Primary school graduate	3.654 (2.273–5.875)	3.547 (2.186–5.755)	3.256 (1.969–5.385)
Middle school graduate	3.059 (2.035–4.596)	3.015 (1.996–4.553)	2.857 (1.866–4.376)
High school graduate	1.553 (1.226–1.967)	1.560 (1.231–1.978)	1.607 (1.261–2.048)
≥University graduate	1	1	1
**Household income**			
Q1: lowest income	1.104 (0.740–1.648)	1.062 (0.709–1.593)	1.096 (0.726–1.656)
Q2: medium-low income	1.021 (0.763–1.366)	1.005 (0.749–1.348)	1.063 (0.792–1.427)
Q3: medium-high income	1	1	1
Q4: highest income	1.371 (1.066–1.763)	1.377 (1.071–1.771)	1.409 (1.091–1.819)

*Q*: quartile; aOR: adjusted odds ratio. Values are shown as aOR (95% confidence intervals).

^a^Model 1: adjusted for age and body mass index.

^b^Model 2: adjusted for covariates included in model 1 plus spousal status, WC, smoking status, drinking status, regular exercise, and stress status

^c^Model 3: adjusted for covariates included in model 2 plus age at menarche and childbirth status.

In models 1, 2, and 3, women with an elementary school education or lower had aORs for irregular menstruation of 3.654 (95% CI, 2.273–5.875), 3.547 (95% CI, 2.186–5.755), and 3.256 (95% CI, 1.969–5.385), respectively, compared to those with a university education or higher. Compared to women with a university education or higher, middle school graduates had aORs of 3.059 (95% CI, 2.035–4.596), 3.015 (95% CI, 1.996–4.553), and 2.857 (95% CI, 1.866–4.376), while high school graduates had aORs of 1.553 (95% CI, 1.226–1.967), 1.560 (95% CI, 1.231–1.978), and 1.607 (95% CI, 1.261–2.048) in models 1, 2, and 3, respectively.

A statistically significant difference in the prevalence of irregular menstruation was observed only for Q4 of household income, with aORs of 1.371 (95% CI, 1.066–1.763), 1.377 (95% CI, 1.071–1.771), and 1.409 (95% CI, 1.091–1.819) in models 1, 2, and 3, respectively.

**Fig 2:**
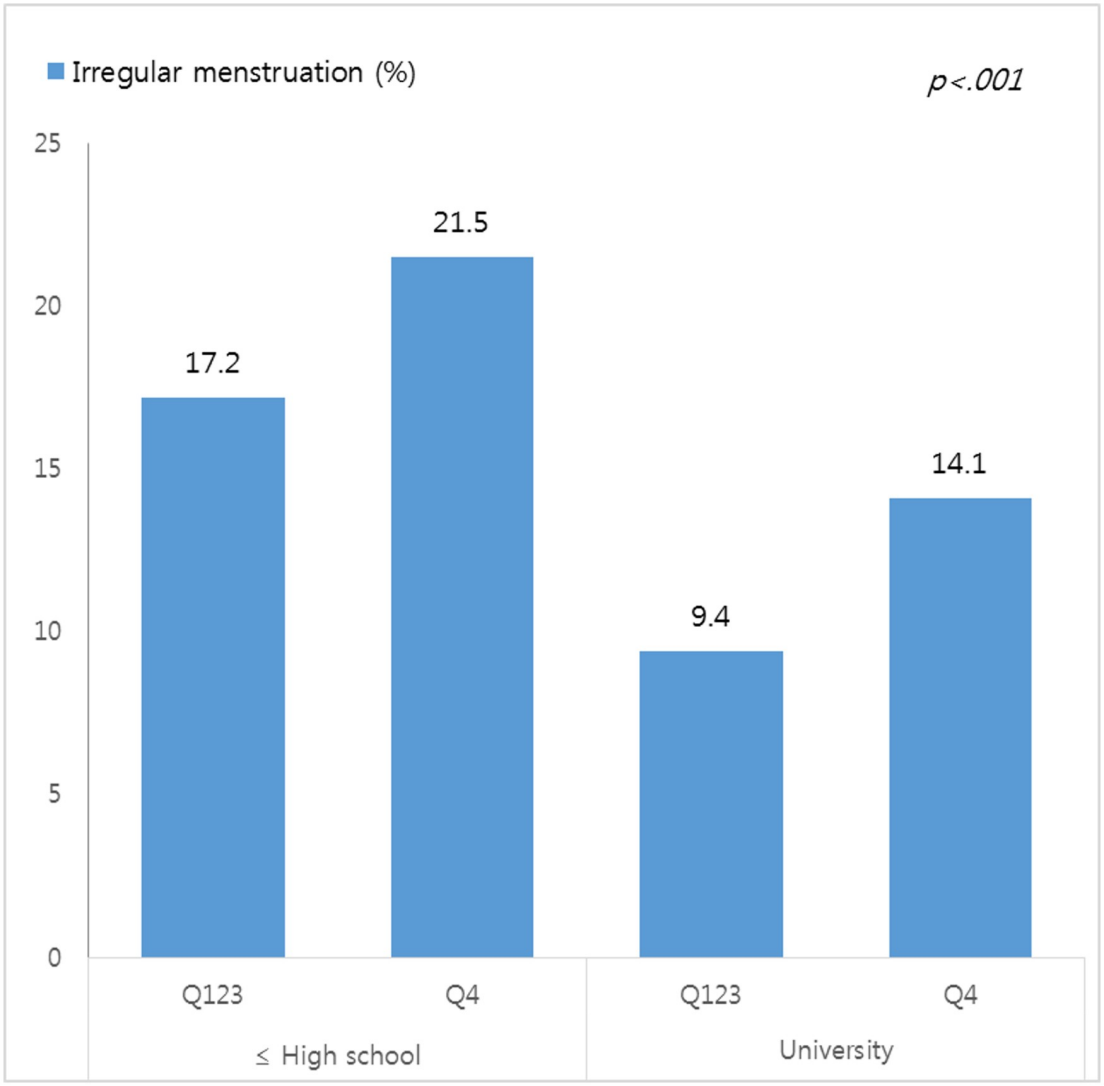
Differences in irregular menstruation by socioeconomic status (n = 669).

Educational level and household income were then combined into four groups for the assessment of group-specific differences in the prevalence of irregular menstruation (Fig 2). Women with a high school education or lower and those in the highest income group had the highest prevalence of irregular menstruation (21.5%). In contrast, women with a university education or higher who reported a medium-high or lower level of income had the lowest prevalence of irregular menstruation (9.4%; *p* <0.001).

## Discussion

In the present study, we investigated the association between the prevalence of irregular menstruation and socioeconomic status among adult Korean women. Lower educational attainment was associated with higher odds of irregular menstruation. Furthermore, women with the highest income (highest quartile) had higher odds of irregular menstruation, after adjusting for educational level. These findings may aid in the development of targeted health improvement programs for women with irregular menstruation based on their socioeconomic status.

According to our results, 14.2% of adult women aged 19–54 years reported having irregular menstruation. In the literature, the prevalence of irregular menstruation varies from 5% to 35.6% depending on age, occupation, and the country of residence [1,2,3,4]. Some reasons for this variability are differences in the definition of irregular menstruation used by researchers and participants’ age distribution. Furthermore, participants may feel shy about discussing menstrual irregularity during a face-to-face interview [20].

In the present study, we observed significant differences in the prevalence of irregular menstruation among adult South Korean women according to spousal status, WC, BMI, smoking status, stress status, age at menarche, and childbirth status. These findings are consistent with those of previous studies, in which significant differences in the prevalence of irregular menstruation were observed according to spousal status [1,13]; WC, BMI, and smoking status [3,7,13,16]; stress [6]; childbirth status; and age at menarche [13,14]. Obese women with high WC and BMI values have elevated levels of insulin and testosterone, and a free androgen index, while the level of sex hormone-binding globulin is decreased, leading to the hormonal changes that cause menstrual irregularity [3,16]. Furthermore, women who smoke cigarettes are at greater risk of experiencing early menopause, menstrual pain, amenorrhea, and irregular menstruation [7,11]. Mental stress also facilitates corticotrophin release, which activates the nervous system and can lead to menstrual problems [4,6,8]. Future studies should examine the individual characteristics that affect stress and hormone secretion, as abnormalities can also lead to irregular menstruation. Combined with previous findings, our results suggest that educational attainment may have a positive impact on women’s health in terms of its effects on obesity, smoking, and mental stress. The complex interplay of health and socioeconomic variables necessitates the creation of individualized reproductive health education programs based on demographic and health-related characteristics.

In general, socioeconomic status and obesity show an inversely proportional association in developed countries [23,24]. In particular, an inverse relationship has been observed between educational level and obesity [23,26,39]. Therefore, obesity in women with a low socioeconomic status can act as a factor promoting irregular menstruation.

The probability of death, self-rated health status, health-related quality of life, and physical and psychological health status are influenced by educational and income levels [22,25,26]. Socioeconomic status is a combined measure of diverse sociological and economic factors; however, educational and income levels have been mainly considered in studies examining health gaps [40]. Education is positively related to long-term health through the reinforcement of mental and social resources (i.e., social support or sense of control) [40]. Therefore, higher education is positively associated with information accessibility and use, the ability to build relationship networks, access to medical services, medical service quality, better housing and occupational environments, and improvements in overall living conditions [2,13,41].

Differences in vocational characteristics or classifications according to educational level have also been observed. High levels of job strain, exhaustion, and stress related to working conditions are known risk factors for gynecologic pain [2,8,12]. Furthermore, there is a higher prevalence of sleep problems among temporary employees and those with a middle school education or lower [42]. Sleep disorders and stress can affect the endocrine system and menstruation [43]. Lower educational levels are likely to result in temporary employment and lead to increased physical tiredness, sleep disorders, and stress, which can subsequently increase the risk for irregular menstruation [2,6,8,40]. Previous studies have shown that stress can occur depending on the level of education and household income, and that subsequent stress-related hormone imbalances can lead to irregular menstruation [8,12].

Working women in high-income households are likely to engage in managerial or specialized positions [9]. Job-related stress may cause sleep problems in professional and managerial workers [42,44]. Physical and mental fatigue, irregular life patterns (such as irregular meal times and working hours), stress, and dietary changes can cause women to feel overwhelmed during their daily lives [42,44]. Furthermore, poor mental health or irregular life patterns can undermine sleep quality, potentially leading to the development of sleep disorders [12,42]. Sleep directly affects ovarian and pituitary gland hormones, and sleep deprivation causes biorhythm abnormality, which can disturb menstruation [42,43]. Although a direct comparison of the association between the prevalence of irregular menstruation and household income is difficult to perform in the absence of longitudinal data, women in high-income households in our study had higher odds of irregular menstruation. Additional studies regarding the cause and effect relationship between irregular menstruation and household income are needed.

Based on our study findings, efforts should be made to educate patients on reproductive health and irregular menstruation, and the importance of early diagnosis and treatment. A major strength of this study is the specific analysis of the association between irregular menstruation in adult women and their socioeconomic status, based on the large size and representativeness of the sample.

This study has several limitations. First, causal relationships could not be determined because this was a cross-sectional study. Therefore, a longitudinal follow-up study is necessary to assess the temporal relationship between socioeconomic factors and menstrual irregularity. Second, we did not include all the factors that are known to be associated with irregular menstruation as our study was based on a secondary analysis of KNHANES V data. Therefore, uncontrolled confounding of our results is possible, further limiting the interpretation of the findings. Third, irregular menstruation includes anovulatory bleeding and ovulatory bleeding; however, we could not obtain information on these through our secondary data analysis. Therefore, a clear definition of irregular menstruation is required for future studies. Despite such limitations, we observed significant associations between the prevalence of irregular menstruation and socioeconomic status.

## Conclusions

Irregular menstruation is an important indicator of current and potential health problems. Thus, it is necessary to evaluate the factors associated with irregular menstruation to determine appropriate preventive and treatment strategies. In the present study, we observed a higher prevalence of irregular menstruation among women with high household incomes and lower educational levels. Public health professionals should recognize the need for early education, detection, and intervention for at-risk populations and stress the importance of building a multidimensional understanding of irregular menstruation.
